# Added value of multifrequency magnetic resonance elastography in predicting pathological grading of pancreatic neuroendocrine neoplasms

**DOI:** 10.1186/s13244-025-02008-3

**Published:** 2025-06-12

**Authors:** Jiaxin Yuan, Jiawei Liu, Tingting Wen, Liqin Wang, Zhenpeng Peng, Ning Zhang, Shi-Ting Feng, Jinhui Yu, Siya Shi, Yanji Luo

**Affiliations:** 1 Department of Radiology, The First Affiliated Hospital, Sun Yat-sen University, Guangzhou, China; 2 Department of Gastroenterology, The First Affiliated Hospital, Sun Yat-sen University, Guangzhou, China

**Keywords:** Magnetic resonance elastography, Pancreatic neoplasms, Carcinoma, Neuroendocrine, Pathological grade

## Abstract

**Objectives:**

To prospectively investigate the pancreatic stiffness (*c*) and fluidity (*φ*) of pancreatic neuroendocrine neoplasms (pNENs), measured using multifrequency magnetic resonance elastography (MRE), and evaluate their performance in predicting pNENs pathological grade.

**Materials and methods:**

This study included 96 untreated patients with pathologically confirmed pNENs who underwent multifrequency MRE within 2 weeks before surgery between September 2021 and November 2023. Independent predictors of pathological grade were identified using multivariate regression analysis, and predictive performance was assessed using receiver operating characteristic curves.

**Results:**

The study included 76 patients with low-grade pNENs (45 men; mean age: 48.7 ± 14.0 years; Grade 1: 34 patients, Grade 2: 42 patients) and 20 patients with high-grade pNENs (10 men; mean age: 54.4 ± 13.8 years; Grade 3: 15 patients, neuroendocrine carcinoma: 5 patients). The two radiologists showed substantial or near-perfect interobserver agreement in evaluating the quantitative parameters. The multivariate regression analysis identified *c* and relative enhancement in the portal venous phase (V) as independent predictors of pathological grade. The combined model (V + *c*) had the best predictive performance (area under the curve (AUC) = 0.930; sensitivity: 95.0%; specificity: 82.9%) and outperformed V (AUC = 0.806, *p* = 0.010), *c* (AUC = 0.847, *p* = 0.021), and *φ* (AUC = 0.709, *p* = 0.003) alone, as well as other clinical and conventional MRI parameters (all *p* < 0.05) in Delong’s test.

**Conclusions:**

Tumour stiffness quantified via multifrequency MRE improved the predictive performance for the pathological grade of pNENs when combined with conventional MRI parameters.

**Critical relevance statement:**

Tumour stiffness quantified using multifrequency magnetic resonance elastography provides a non-invasive, preoperative method for predicting the pathological grade of pancreatic neuroendocrine neoplasms. Predictive performance improves when combined with conventional MRI parameters, facilitating clinical decision-making and prognostic prediction.

**Key Points:**

- Multifrequency magnetic resonance elastography (MRE) can indicate stiffness and fluidity of pancreatic neuroendocrine neoplasms (pNENs).
- Tumour stiffness combined with conventional MRI parameters can independently predict pNENs pathological grade.
- Multifrequency MRE can serve as a biomarker for the prediction of pNENs pathological grade.

**Graphical Abstract:**

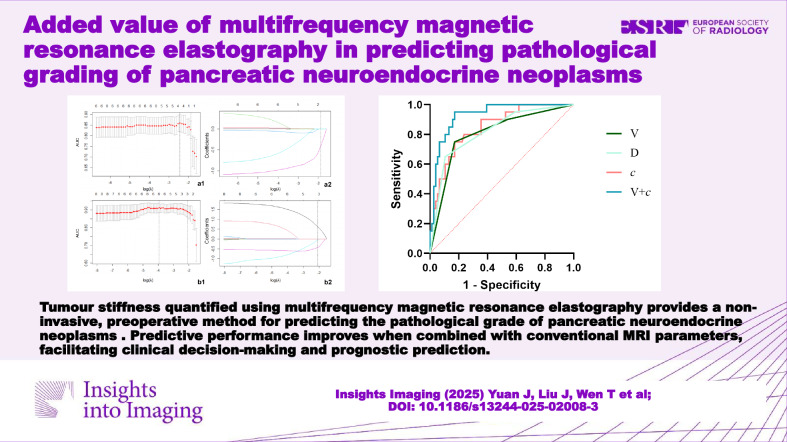

## Introduction

Pancreatic neuroendocrine neoplasms (pNENs) encompass a spectrum ranging from well-differentiated neuroendocrine tumours (NETs) to poorly differentiated neuroendocrine carcinomas (NECs) [1]. These two entities differ in clinical manifestations, epidemiological data, histological features, and genetic profiles. The 2017 and 2019 WHO classifications provide updated diagnostic categories and criteria, classifying NETs into NET-G1, NET-G2, and NET-G3. NECs encompass poorly differentiated NENs and mixed neuroendocrine non-neuroendocrine carcinomas [2].

Tumour grading and differentiation are key determinants of clinical behaviour and lead to different lymph node dissection area, prognoses and treatment options [3]. The 5-year survival rates for G1, G2 and G3 were 77.33%, 63.06% and 20.04%, respectively [4]. However, the survival of patients with pancreatic NECs is usually less than 1 year [5]. The treatment options for pNENs must be considered based on the size of the primary lesion, local involvement, lymph node metastasis and distant metastasis [6–8]. Radical surgical resection is the prevailing treatment for localised NETs and NECs. However, for localised NECs, despite surgery, many patients continue to have rapid disease progression and a high propensity for metastatic dissemination [7]. The use of neoadjuvant therapy for NECs has been shown to result in a downgrading of staging, an increase in the rate of complete resection, and a lower risk of local and systemic recurrence [7]. Furthermore, the 2023 ENETs guidelines refer to the use of preoperative peptide receptor radionuclide therapy (PRRT) for somatostatin receptor (SSTR) positive in locally advanced or oligometastatic G1 and G2 nonfunctioning-pNENs (NF-pNENs) [6]. Therefore, accurate pNENs grading is crucial for clinical decision-making.

Histological examination is the gold standard for grading and differentiating pNENs [2, 9]. However, as an invasive examination, biopsy can lead to serious complications, and the high heterogeneity of pNENs may cause variations in the pathological results [10, 11]. In applying CT for grading pNENs, studies have discovered that, compared with NETs, NECs prefer to invade adjacent structures and develop distant metastases [12]. Decreased tumour perfusion and hypo-enhancement patterns correlate with poorly differentiated NENs [13, 14]. However, NET-G2/G3 may also show reduced perfusion [15]. PET/CT or PET/MRI can diagnose well-differentiated pNENs owing to high somatostatin receptor expression; however, these examinations are costly and pose radiation concerns [16].

Multifrequency magnetic resonance elastography (MRE) has emerged as a novel and promising non-invasive method for distinguishing benign from malignant tumours and predicting tumour invasion by exploring the mechanical properties of tumour tissues [17–19]. Compared to 2D MRE, multifrequency MRE captures the viscoelastic properties of tissues more comprehensively by simultaneously utilising data from multiple single frequencies in three dimensions and combining them with post-processing algorithms that are resistant to graphical noise interference [20, 21]. This approach facilitates the alignment of the recovered parameters with the true physical properties of the tissue, thereby producing a more nuanced representation of the tissue structure [22]. This, in turn, is crucial for accurately assessing the physiological and pathological state of the tissue. Multifrequency MRE provides high-resolution elastography and can be used in the routine examination of various organ tissues, where it can quantitatively measure pancreatic stiffness and fluidity [18–20, 23]. The fibrosis level and the presence of cell adhesion affect tumour stiffness and fluidity [17, 19, 24]. High-grade pNENs exhibit more significant malignant biological behaviour, causing changes in cell adhesion, which can be used to predict tumour grade and differentiation [25].

Therefore, we suppose that multifrequency MRE parameters may enhance the preoperative diagnostic precision in differentiating between low- and high-grade pNENs, ultimately aiding clinical decision-making and personalised treatment planning.

## Materials and methods

### Study population

This single-centre, prospective study was approved by the ethics committee of the First Affiliated Hospital of Sun Yat-sen University, and written informed consent was obtained from all patients. Inclusion criteria involved patients with pathologically confirmed pNENs who underwent pancreatic multifrequency MRE examination within 2 weeks before surgery, between September 2021 and November 2023, without prior treatment. The pNENs were graded and staged based on the WHO 2017 classification criteria: NET-G1, NET-G2, NET-G3, and NEC categories.

**Fig. 1:**
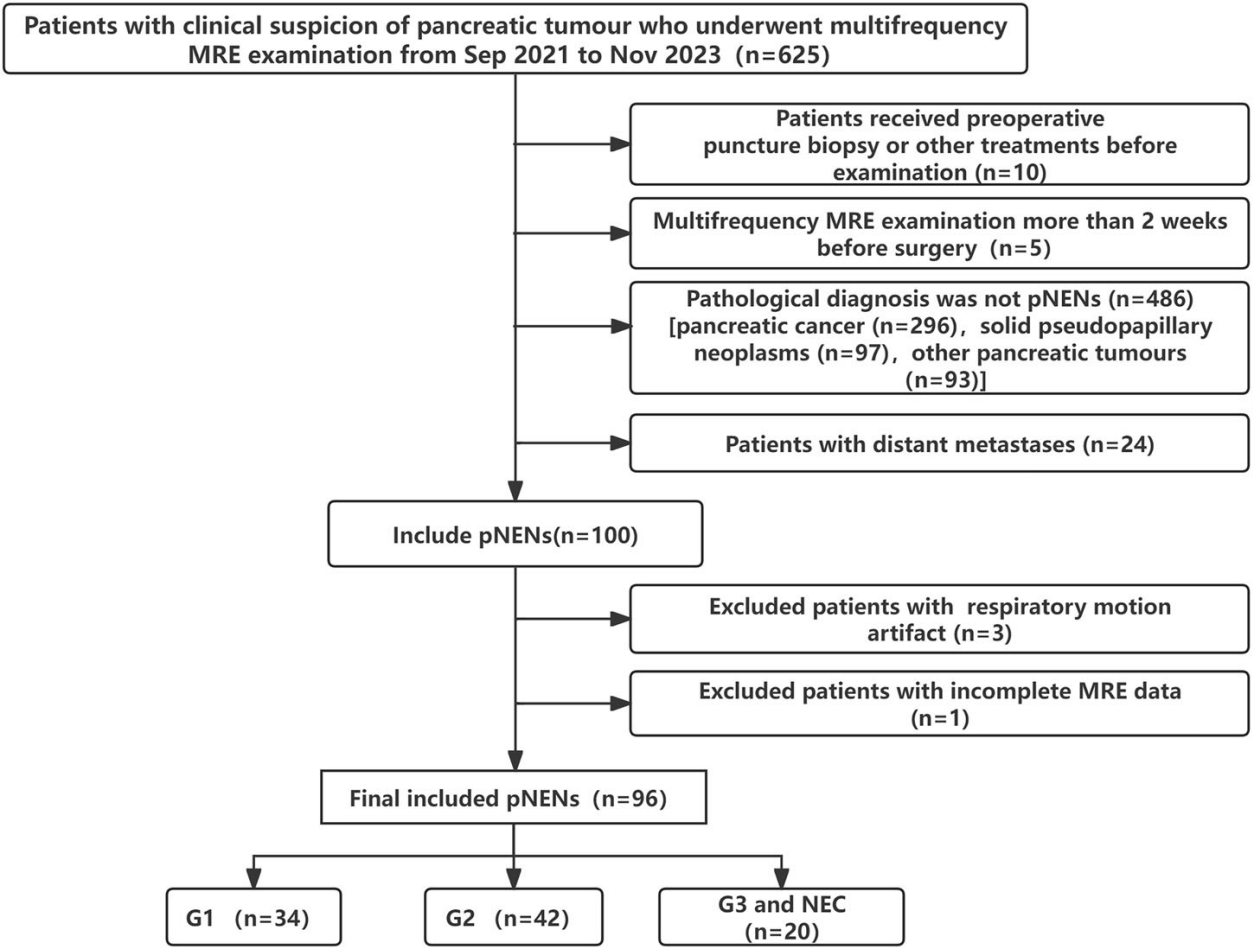
Flowchart of the study. MRE, magnetic resonance elastography; pNENs, pancreatic neuroendocrine neoplasms; G1/2/3, Grade-1/2/3; NEC, neuroendocrine carcinoma

Exclusion criteria included patients who were unable to cooperate during the examination and caused respiratory motion artefacts and those with incomplete MRE data. The specific flowchart is shown in Fig. 1.

### Imaging technique

#### MR sequences and multifrequency MRE

Patients were examined in the supine position after 6–8 h of fasting and dehydration. Multifrequency MRE was performed using a 3-Tesla MRI system (MAGNETOM Prisma; Siemens Healthcare), with an 18-channel phased-array surface coil. Details of the MRI parameters are presented in Table 1. The arterial phase is obtained 20–35 s after injection of contrast agent (Gadobutrol Injection; Bayer AG; 0.1 mL/kg), while the portal venous phase and delayed phase are obtained 60 s and 3 min later [26]. And the protocol information can be found in the supplementary material.

**Table 1:** Sequence parameters of the pancreatic MRI protocol

Sequence	Plane	Acquisition	TR/TE (ms)	Section thickness (mm)	FOV (mm)	FA (°)	Acquisition time (s)
T1-VIBE-Dixon	Axial	BH	3.97/1.29–2.52	2	380	9	21
T2-HASTE	Axial	BH	1200 /78	4	380	160	31
T2-trigger-tse-fs	Axial	NT	2000/77	4	380	103	180–300
VIBE-q-dixon (6 Echos)	Axial	BH	9.00/1.05–7.38	2	426	4	20
DWI	Axial	FB	3700/47	5	380	-	76
DCE	Axial	BH	2.75/1.05	2	380	12.5	25–30/60/180
MRE	Axial	FB	4140/69	2	256	90	431

*TR* repetition time, *TE* echo time, *FOV* field of view, *FA* flip angle, *VIBE* volume-interpolated breath-hold, *HASTE* half-Fourier acquisition single-shot turbo spin-echo, *NT* navigator triggered, *BH* breath-hold, *FB* free-breathing, *tse* turbo spin-echo, *fs* fat-saturated, *DWI* diffusion-weighted imaging, *DCE* dynamic contrast-enhanced, *MRE* magnetic resonance elastography

Multifrequency MRE was conducted during free-breathing before contrast administration using four drive frequencies of 30, 40, 50 and 60 Hz, induced by two compressed-air drivers (8.0 × 4.0 × 1.0 cm) positioned anteriorly and posteriorly on the surface projection of the pancreas [23]. Multifrequency wave field data were acquired in 7 min and 22 s using a single-shot spin-echo EPI sequence with flow-compensated motion-encoding gradients (MEGs) and an amplitude of 45 mT/m. The MEG frequencies were 37.20 Hz, 37.20 Hz, 37.48 Hz, and 44.88 Hz for the vibration frequencies of 30.00 Hz, 40.00 Hz, 50.00 Hz, and 60.00 Hz, respectively. The MEG data in our study were collected with three directional measurements. Additionally, the phase-off set number was established at 8.

#### Multifrequency MRE data post-processing

MRE data were processed using the publicly available multifrequency MRE pipeline at https://bioqic-apps.com [22]. Multifrequency MRE obtains spatial gradients through corresponding inversion algorithms and applies that in 2D and spatial derivatives, thereby achieving a reliable measurement of small lesions [20]. It produces high-resolution elastography parameters, generating full field-of-view high-spatial resolution maps of shear wave speed (*c*) and the loss angle of the complex shear modulus (*φ*) [20]. As *c* directly correlates with the magnitude of the complex shear modulus, it can be considered a surrogate marker of stiffness [27]. The loss angle of the complex shear modulus (*φ*) is related to the fluidity properties of the tissue, ranging from 0 to π/2, marking the fluidity transition from solid to fluid [19]. Previous studies have indicated that the values of pancreatic stiffness and fluidity in healthy individuals in this region are 1.456 m/s and 0.830 rad, respectively [27]. To facilitate comparison with previous findings, shear wave speed and phase angle are used to refer to *c* and *φ* when describing quantitative data in the results in our research, while stiffness and fluidity represent qualitative data.

### Image evaluation

Two radiologists with 8 (Reader 1) and 15 (Reader 2) years of experience in abdominal imaging diagnosis evaluated patient image data. They manually drew three regions of interest (ROIs) of at least 100 mm^2^ at the largest slice of the solid portion of the tumour and two adjacent slices without calcifications, blood vessels, necrotic and cystic areas, or pancreatic duct dilation. The mean values of each ROI were recorded to obtain overall measurements for each lesion. Readers 1 and 2, blinded to the histopathological and clinical results, used Image J software (version 1.5; National Institutes of Health, Bethesda, MD, USA) to measure the stiffness and fluidity of all patients, and the measurement was repeated by Reader 1 one month later (Reader 1A). The mean values of each ROI were recorded to obtain overall measurements for each lesion. Two weeks after the initial measurement, the two radiologists independently evaluated other radiological features in conventional MR sequences, including (1) tumour location (head-neck or body-tail), (2) tumour size (maximum tumour diameter) [28], (3) tumour components, (4) necrosis, (5) intratumoural haemorrhage, (6) apparent diffusion coefficient (ADC) value (b = 800 s/mm^2^), and (7) enhancement pattern. Details of the assessment can be found in the supplementary material.

### Clinical and pathological characteristics

A clinician not involved in the radiological feature evaluation recorded clinical features, such as age, sex, neuron-specific enolase (NSE) levels, and carbohydrate antigen 19-9 (CA19-9) levels. Two pathologists with 5 years of diagnostic experience jointly performed tumour-specific immunohistochemical testing of tumour specimens within 7 days after surgery and assessed the histological grading according to mitotic rate and Ki-67 index [1]. Histopathological grading of pancreatic neuroendocrine neoplasms was performed according to the 2017 WHO classification of tumours. All pathologists were blinded to imaging results, and immunohistochemical markers such as chromogranin A and synaptophysin were employed to differentiate histological grade. Two pathologists with 5 years of diagnostic experience jointly performed hematoxylin-eosin staining and tumour-specific immunohistochemical testing of tumour specimens within 7 days after surgery. Specific immunohistochemical techniques and indicators are detailed in the supplementary material. If the assessment results were disputed, a third pathologist with 22 years of clinical experience conducted the assessment and documented the final results. In our research, low-grade pNENs included G1 and G2, whereas high-grade pNENs included G3 and NEC.

### Statistical analysis

Using the Shapiro–Wilk test for normal distribution [5], continuous variables are expressed as mean ± standard deviation or median (interquartile range) and compared using Student’s *t*-test or Kruskal–Wallis test. Categorical data are expressed as frequencies or percentages and compared using the chi-square or Fisher’s exact test. Inter/Intra-observer agreements were assessed via the Bland–Altman analysis, intraclass correlation coefficients (ICCs), and Kappa values (κ), graded into five categories: 0–0.20, slight; 0.21–0.40, fair; 0.41–0.60, moderate; 0.61–0.80, substantial; and 0.81–1.00, near-perfect [29, 30]. All characteristics were included in the univariate logistic analysis, the resulting characteristics were first screened with LASSO regression analysis before being subjected to multivariate logistic regression analysis. Receiver operating characteristic (ROC) curve analysis was performed to determine and compare the diagnostic performances of multifrequency MRE and conventional MRI. The area under the curve (AUC), sensitivity, and specificity were calculated, and the cutoff values were selected based on Youden’s index. AUCs were compared using Delong’s test. Statistical analysis was performed using SPSS (version 24.0; SPSS Inc.), MedCalc software (MedCalc Software Ltd.), and GraphPad Prism (version 5.01, GraphPad Prism for Windows). Statistical significance was defined as *p* < 0.05.

## Results

### Patient and lesion characteristics

**Fig. 2:**
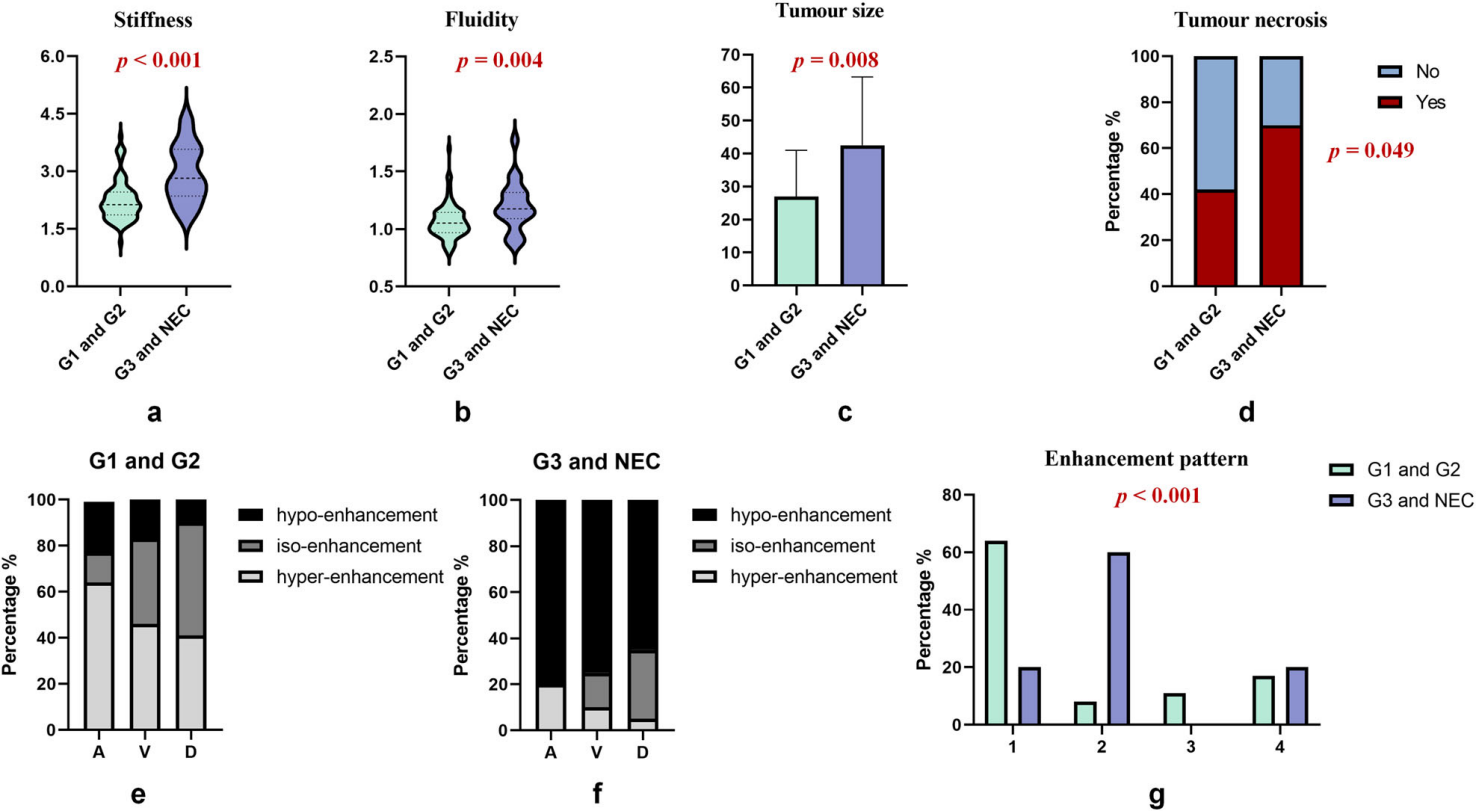
Statistical charts of relevant radiological features. **a**, **b** Violin plots showing the stiffness (**a**) and fluidity (**b**) of low- and high-grade pancreatic neuroendocrine neoplasms. **c**, **g** Bar graphs representing tumour size (**c**) and enhancement patterns (**g**). **d**–**f** Scaled bar graphs illustrating intratumoural necrosis (**d**) and tumour enhancement (**e**, **f**). G1/2/3, Grade-1/2/3; NEC, neuroendocrine carcinoma

**Fig. 3:**
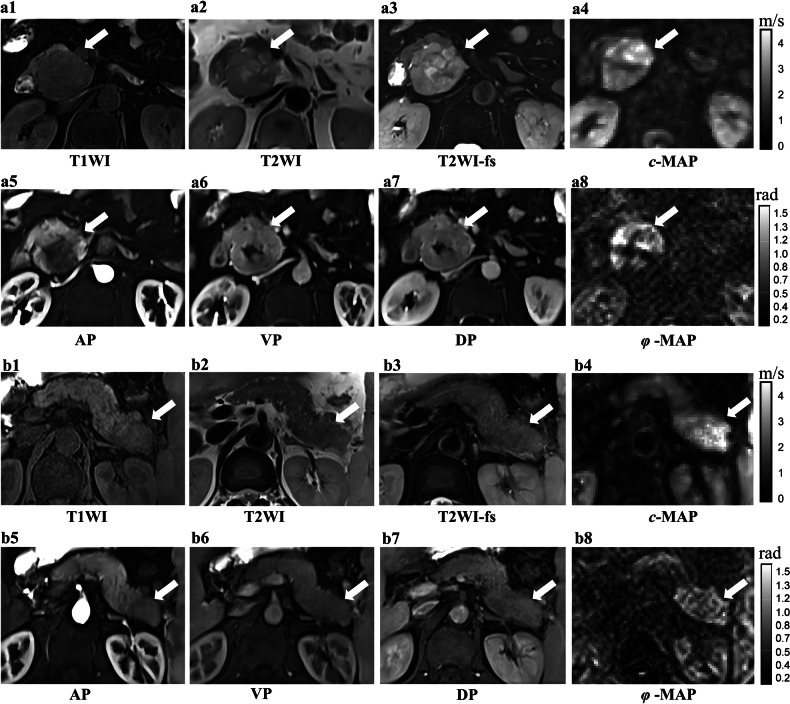
Clinical cases of high- and low-grade pNENs. **a1**–**8** A 66-year-old woman diagnosed with a pNEN (G2). **a1**–**3** Axial T1WI, T2WI, and T2WI-fs images showing a solid lesion in the pancreatic head (arrow). **a5**–**7** Dynamic enhancement images showing a type 3 enhancement pattern (iso-enhancement in all three phases). **a4**, **a8** *c* = 2.29 m/s; *φ* = 1.03 rad. **b1**–**8** A 52-year-old man diagnosed with a pNEN (G3). **b1**–**3** Axial T1WI, T2WI, and T2WI-fs images showing a solid lesion in the pancreatic tail (arrow). **b5**–**7** Dynamic enhancement images showing a type 4 enhancement pattern (hypo-enhancement in the arterial and portal venous phases compared with pancreatic parenchyma, with iso-enhancement in the delayed phase). **b4**, **b8** *c* = 3.24 m/s; *φ* = 1.88 rad. pNENs, pancreatic neuroendocrine neoplasms; G1/2/3, Grade-1/2/3; T1WI, T1-weighted imaging; T2WI, T2-weighted imaging; fs, fat-saturated; *c*, shear wave speed (stiffness); *φ*, phase angle (fluidity); AP, arterial phase; VP; portal venous phase; DP, delayed phase

This study enrolled 96 patients with pNENs, divided into two groups: 76 patients with low-grade pNENs (45 men; mean age: 48.7 ± 14.0 years) and 20 patients with high-grade pNENs (10 men; mean age: 54.4 ± 13.8 years). Table 2 and Fig. 2 present the general characteristics of the enrolled patients. Patients with low-grade pNENs had smaller tumour sizes (27.0 mm vs. 42.5 mm, *p* = 0.008) and less frequent intratumoural necrosis than did those with high-grade pNENs (*p* = 0.049). Low-grade pNENs more frequently exhibited hyper-enhancement in the arterial, portal, and delayed phases than did high-grade pNENs, with enhancement patterns differentiating the two groups (all *p* < 0.001). The median shear wave speed (*c*) (2.13 m/s vs. 2.82 m/s; *p* < 0.001) and phase angle (*φ*) (1.05 rad vs. 1.18 rad; *p* = 0.004) values were lower in low-grade pNENs. Age, sex, NSE levels, CA19-9 levels, symptoms, tumour location, tumour components, intratumoural haemorrhage, and ADC value did not significantly differ between the two groups. Figure 3 illustrates cases with different grades of pNENs. Examples of actual wave images are given in the supplementary material.

**Table 2:** Baseline characteristics of patients and the univariate analysis of different features in pNENs

Variables	G1 and G2 (*n* = 76)	G3 and NEC (*n* = 20)	*p*-value^†^	Univariate analysis	
				OR (95% CI)	*p-*value
Age (years)	48.7 ± 14.0	54.4 ± 13.8	0.11	1.03 (0.99, 1.07)	0.11
Sex (*n*, %)			0.63	1.45 (0.54, 3.90)	0.46
Male	45 (59)	10 (50)	-		
Female	31 (41)	10 (50)	-		
NSE (ng/mL)	19.9 (16.0, 28.0)	37.6 (14.2, 112.3)	0.21	1.01 (1.00, 1.02)	0.03*
CA19-9 (U/mL)	8.8 (4.3, 20.0)	16.6 (7.0, 34.7)	0.10	1.00 (0.99, 1.01)	0.56
Symptoms (*n*, %)					
Asymptomatic	29 (38)	6 (30)	0.68	0.69 (0.24, 2.01)	0.50
Abdominal pain or distension	30 (39)	9 (45)	0.85	1.25 (0.46, 3.39)	0.66
Hypoglycemia	9 (12)	0 (0)	0.20	0 (0, Inf)	0.99
Other symptoms (e.g., vomiting, malaise)	8 (11)	5 (25)	0.14	2.83 (0.81, 9.88)	0.10
Grade of differentiation*, n* (%)					
G1	34 (45)	-	-		
G2	42 (55)	-	-		
G3	-	15 (75)	-		
NEC	-	5 (25)	-		
Tumour location			0.78	0.77 (0.29, 2.06)	0.60
Head and neck	33 (43)	10 (50)	-		
Body and tail	43 (57)	10 (50)	-		
Tumour size (mm)	27.0 (17.0, 41.0)	42.5 (24.7, 59.7)	0.008*	1.04 (1.01, 1.06)	0.003*
Tumour components			1.00	1.03 (0.61, 1.72)	0.92
Predominantly solid	48 (63)	13 (65)	-		
Solid, cystic	27 (36)	7 (35)	-		
Predominantly cystic	1 (1)	0 (0)	-		
Necrosis	32 (42)	14 (70)	0.049*	3.21 (1.11, 9.25)	0.03*
Haemorrhage	13 (17)	3 (15)	1.00	0.85 (0.22, 3.35)	0.82
ADC (× 10^−^^3^ mm^2^/s)	1064.1 (948.5, 1198.3)	955.9 (866.1, 1077.4)	0.23	0.99 (0.99, 1)	0.09
Enhancement pattern			< 0.001*	1.32 (0.88, 1.98)	0.18
Type 1	49 (64)	4 (20)	-		
Type 2	6 (8)	12 (60)	-		
Type 3	8 (11)	0 (0)	-		
Type 4	13 (17)	4 (20)	-		
Type 5	0 (0)	0 (0)	-		
A*, n*%			< 0.001*	0.27 (0.14, 0.52)	< 0.001*
hypo	17 (22)	16 (80)	-		
iso	10 (13)	0 (0)	-		
hyper	49 (64)	4 (20)	-		
V*, n*%			< 0.001*	0.18 (0.07, 0.42)	< 0.001*
hypo	13 (17)	15 (75)	-		
iso	28 (37)	3 (15)	-		
hyper	35 (46)	2 (10)	-		
D*, n*%			< 0.001*	0.12 (0.04, 0.32)	< 0.001*
hypo	8 (10)	13 (65)	-		
iso	37 (49)	6 (30)	-		
hyper	31 (41)	1 (5)	-		
Shear wave speed (m/s)	2.13 (1.88, 2.45)	2.82 (2.36, 3.56)	< 0.001*	5.00 (2.16, 11.53)	< 0.001*
Phase angle (rad)	1.05 (0.98, 1.14)	1.18 (1.10, 1.31)	0.004*	1.50 (1.12, 2.01)	0.006*

The bold values are those that exhibited statistical differences in the statistical analysis

The five enhancement patterns are explained in detail as follows: hyper-enhancement in the arterial phase (Type 1) and persistent hypo- or iso-enhancement in all three phases (Types 2 and 3). Arterial phase with hypo- or iso-enhancement included portal hypo-enhancement with gradual delayed hyper-enhancement on delayed phase images (Type 4) and portal hyper-enhancement with hyper- or iso-enhancement on delayed phase images (Type 5)

Continuous variables are presented as means ± standard deviation or median (interquartile range), and categorical variables are presented as numbers (percentages)

*OR* odds ratio, *CI* confidence interval, *pNENs* pancreatic neuroendocrine neoplasms, *NSE* neuron, specific enolase, *CA19-9* carbohydrate antigen 19-9, *NEC* neuroendocrine carcinoma, *ADC* apparent diffusion coefficient, *A* relative enhancement degree of lesions during the arterial phase, *V* relative enhancement degree of lesions during the portal venous phase, *D* relative enhancement degree of lesions during the delayed phase

^†^ Continuous variables are compared using Student’s *t*-test or Kruskal–Wallis test, and categorical data are compared using the chi-square or Fisher’s exact test

* Statistically significant (*p* < 0.05)

### ICCs of multifrequency MRE

**Fig. 4:**
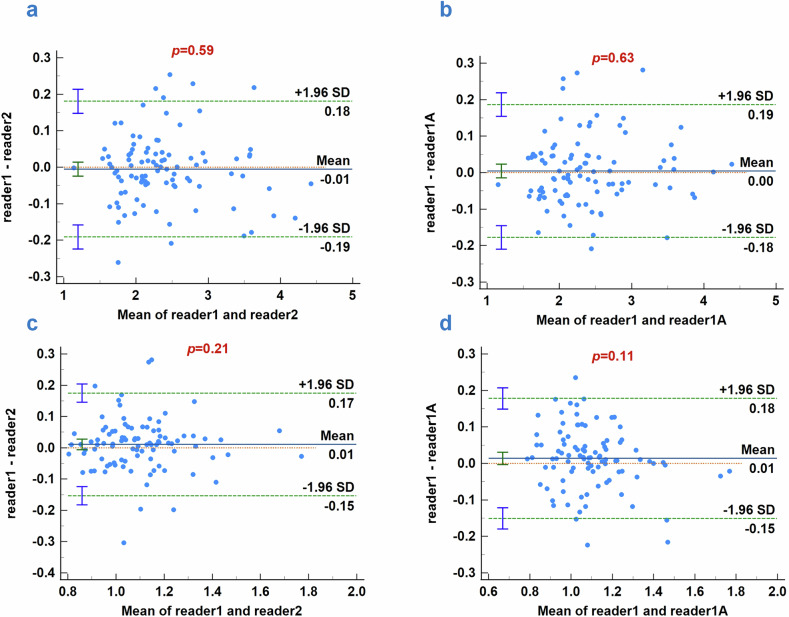
Bland–Altman analyses of reader agreement. Reader 1: First measurement of the images. Reader 1A: Second measurement of the images after 1 month. Reader 2: Measurement of the images. **a**, **b** Bland–Altman analyses of reader agreement for pancreatic stiffness; **c**, **d** Bland–Altman analyses of reader agreement for pancreatic fluidity

The Bland–Altman analyses showed near-perfect agreements between the two readers for measurements of pancreatic shear wave speed (*c*) (Fig. 4a, b) and phase angle (*φ*) (Fig. 4c, d). The ICCs demonstrated near-perfect agreement for *c* (0.989, 95% confidence interval (CI): 0.984–0.993, *p* < 0.001) and *φ* (0.887, 95% CI: 0.835–0.923, *p* < 0.001). Moderate to near-perfect agreements were observed for the enhancement pattern (κ = 0.932, *p* < 0.001). The ICCs and kappa values of other features are provided in the supplementary material.

### Relevant features for predicting the pathological grade of pNENs

**Fig. 5:**
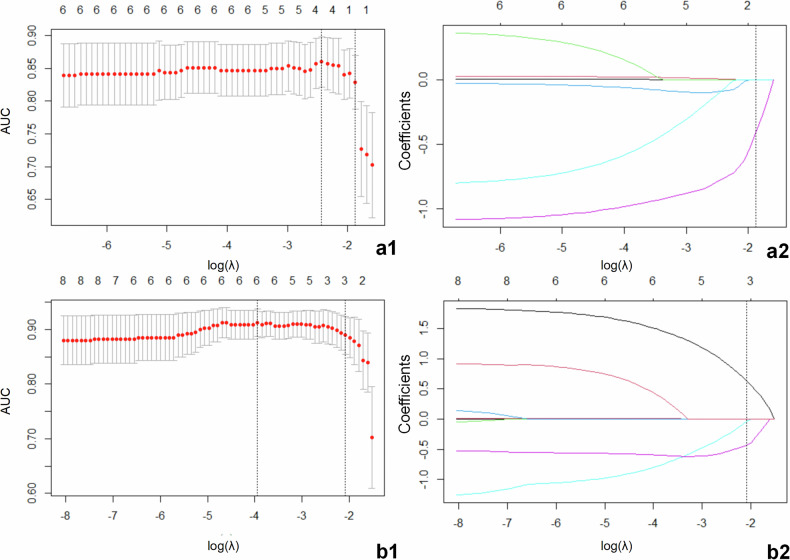
Feature selection for LASSO regression. **a1**, **b1** Selection of the tuning parameter (λ) for LASSO regression. The area under the curve (AUC) was plotted against log(λ), and fivefold cross-validation was used to select the optimal value of λ (0.153) in the **a1** model and λ (0.124) in the **b1** model. **a2**, **b2** LASSO coefficient profiles of features, with each coloured line representing the coefficient for each feature. A vertical black line is plotted at the selected log(λ) (−1.875) and log(λ) (−2.085), respectively, where the non-zero coefficients were obtained with one (D) in the non-MRE parameters and three (V, D, c) in all parameters. **a1**, **a2** Non-MRE parameters. **b1**, **b2** All parameters. LASSO, least absolute shrinkage and selection operator; V, relative enhancement degree of lesions during the portal venous phase; MRE, magnetic resonance elastography; D, relative enhancement degree of lesions during the delayed phase; *c*, shear wave speed (stiffness)

Univariate analysis of clinical and radiological features showed that NSE (*p* = 0.03; odds ratio (OR), 1.01; 95% CI: 1.00–1.02), tumour size (*p* = 0.003; OR, 1.04; 95% CI: 1.01–1.06) and necrosis (*p* = 0.03; OR, 3.21; 95% CI: 1.11–9.25), relative enhancement degree in the arterial phase (A) (*p* < 0.001; OR, 0.27; 95% CI: 0.14–0.52), relative enhancement degree in the portal venous phase (V) (*p* < 0.001; OR, 0.18; 95% CI: 0.07–0.42), and relative enhancement degree in the delayed phase (D) (*p* < 0.001; OR, 0.12; 95% CI: 0.04–0.32), shear wave speed (*c*) (*p* < 0.001; OR, 5.00; 95% CI: 2.16–11.53), and phase angle (*φ*) (*p* = 0.006; OR, 1.50; 95% CI: 1.12–2.01) were associated with the prediction of pathological grading of pNENs (Table 2). And we conducted a multicollinearity analysis among these eight significant features in the univariate logistic analysis. The results showed that none of the above eight features are covariant, implying that they are not correlated with each other (Table S1). Three parameters of V, D, and *c* were screened by LASSO regression analysis for the parameters that were significant in the above univariate analysis (Fig. 5). The above three parameters were then included in a multivariate regression analysis, and only V (*p* < 0.001; adjusted OR, 0.18; 95% CI: 0.07–0.47) and *c* (*p* < 0.001; adjusted OR, 9.23; 95% CI: 2.80–30.42) were independent predictors of the pathological grade of pNENs.

### Performance of relevant clinical and radiological features and the combined model

**Fig. 6:**
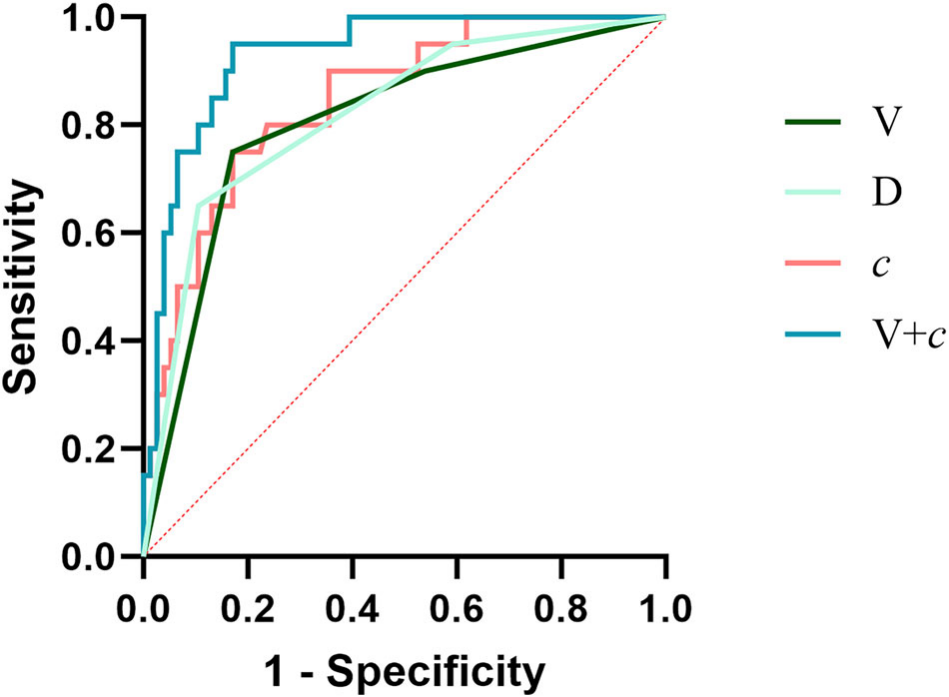
Receiver operating characteristic curves. The combined model (V + *c*) shows the highest predictive performance (AUC = 0.930) compared with that of other radiological (V, D and *c*) features (All *p* < 0.05). V, relative enhancement degree of lesions during the portal venous phase; D, relative enhancement degree of lesions during the delayed phase; *c*, shear wave speed (stiffness)

We evaluated the three parameters screened by LASSO regression analysis for predicting the pathological grade of pNENs: *c* (AUC, 0.847; 95% CI: 0.756–0.938; cutoff, 2.57 m/s), V (AUC, 0.806; 95% CI: 0.699–0.912) and D (AUC, 0.821; 95% CI: 0.724–0.919). The results of the diagnostic performance of the other parameters in the univariate analysis are detailed in the supplementary material (Table S2 and Fig. S1). The two parameters derived from the multivariable regression analysis were combined into a model (V + *c*), and the equation for the combined model (V + *c*) is “predicted value = −4.06 − 1.70*V + 2.22*c”. The combined model (V + *c*) (AUC, 0.930; 95% CI: 0.876–0.984) demonstrated the best predictive performance compared with that of *c* (*p* = 0.021), V (*p* = 0.010), D (*p* = 0.018), respectively. (Table 3 and Fig. 6).

**Table 3:** Performance of radiological features and the models for differentiating low-/high-grade pNENs

Variables	Delong’s test	AUC (95% CI)	Ac	Se	Sp	PPV	NPV	Cutoff value
V	**0.010**^a^	0.806 (0.699–0.912)	0.812	0.750	0.829	0.536	0.926	-
D	**0.018**^a^	0.821 (0.724–0.919)	0.844	0.650	0.895	0.619	0.907	-
*c* (m/s)	**0.021**^a^	0.847 (0.756–0.938)	0.812	0.750	0.829	0.536	0.926	2.57
V + *c*	-	0.930 (0.876–0.984)	0.854	0.950	0.829	0.594	0.984	-

The bold values are those that exhibited statistical differences in Delong’s test

The LASSO regression analysis yielded one (D) in the non-MRE parameters and three (V, D, *c*) in all parameters. Incorporating V, D and *c* into the multivariate regression analysis resulted in only V and *c*

*pNENs* pancreatic neuroendocrine neoplasms, *AUC* area under the curve, *CI* confidence interval, *Ac* accuracy, *Se* sensitivity, *Sp* specificity, *PPV* positive predictive value, *NPV* negative predictive value, *NSE* neuron-specific enolase, *V* relative enhancement degree of lesions during the portal venous phase, *D* relative enhancement degree of lesions during the delayed phase, *c* shear wave speed (stiffness)

^a^ Statistically significant (*p* < 0.05)

## Discussion

The pathological grading of pNENs is crucial for determining clinical treatment strategies and prognosis [1]. Our results indicated that multifrequency MRE parameters improved the predictive efficacy of conventional MRI parameters in determining the pathological grading of pNENs. Compared with other parameters, the combined model constructed using stiffness (*c*) and relative enhancement degree of lesions during the portal venous phase (V) demonstrated the highest predictive performance.

Multifrequency MRE is a promising non-invasive method with unique advantages over single-frequency MRE for small organs and retroperitoneal organs such as the pancreas. This is because the vibrational waves of the multifrequency MRE are efficiently transmitted to the organ, ensuring resolution and improving measurement accuracy [20]. In addition, multifrequency MREs are able to reproduce rich organisational detail and reduce image distortion by combining it with post-processing algorithms that are resistant to graphical noise interference [21]. Moreover, our results showed that the measurement of multifrequency MRE parameters of pancreas by radiologists with different seniority showed excellent agreements, and the results could be reproduced by repeated scans at short interval in a previous study [27], which proved the reliability of multifrequency MRE equipment in the application of pancreatic diseases. The mechanical characteristics of tumours provide important diagnostic information [31]. Multifrequency MRE can quantitatively measure pancreatic stiffness and fluidity, reflecting the pathological characteristics of lesions to some extent. Some studies have demonstrated a positive correlation between the degree of fibrosis and tissue stiffness [32]. Furthermore, NETs have been shown to be typically associated with fibrosis [33], and pNENs with extensive fibrotic features have been identified as being aggressive [34, 35]. The more aggressive pNENs have higher stiffness [18]; this is similar to our findings that G3 and NEC are more aggressive and have more pronounced fibrotic features as well as high stiffness values. Gao et al demonstrated that NEC exhibits a tumour microenvironment comparable to that observed in pancreatic ductal adenocarcinoma (PDAC) [36]. PDAC is characterised by a distinctive proconnective tissue proliferative tumour microenvironment, resulting in substantial tissue fibrosis [37]. The proconnective tissue proliferative stroma impairs immune cells, tumour blood perfusion, and oxygen delivery, which hinders angiogenesis [38]. This results in a reduction in local blood perfusion of the lesion, which is consistent with our observation that, in comparison to G1 and G2, G3 and NEC exhibit distinctive hypo-enhancement relative to the pancreatic parenchyma in the arterial, portal venous, and delayed phases. Our study analysed the differences in overall stiffness using multiple measurements of tumour entities, demonstrating the feasibility of future non-invasive quantitative analyses of fibrosis levels in pNENs.

G3 and NEC in our study had higher fluidity than did G1 and G2. Previous studies have reported that fluidity correlates with the aggressiveness of malignant tumours, as it reflects the ability of cells to adhere to the tumour tissue [19]. More aggressive tumours are more likely to invade surrounding tissues and accumulate more hydrophobic, disorganised proteins, which increase tissue mechanical friction, resulting in higher fluidity [31]. G3 and NEC display more pronounced malignant biological behaviour than did G1 and G2, exhibiting higher invasiveness and fluidity. Although fluidity, another superior indicator of multifrequency MRE, was not ultimately included in the combined model, fluidity performed superiorly in the univariate analysis. Previous studies have shown that during cancer progression, invasive and metastatic tumour cells may adaptively soften to facilitate tumour migration through narrow tissue spaces [39]. It is reasonable to predict that fluidity has greater potential to discriminate between G3 and NEC, but due to the small sample size of G3 and NEC that can be included, there is no way to further segmentation on this basis, and we will explore this aspect further in the future.

Tumour enhancement patterns were helpful in predicting the pathological grade of pNENs, and V was identified as an independent predictor. This is consistent with the two previous studies on dynamic enhanced CT, which showed that the degree of enhancement during the portal vein phase is an effective sign to differentiate between well- and poorly differentiated pNENs [12, 40]. In our study, G3 and NEC exhibited hypo-enhancement in the arterial, portal venous, and delayed phases compared with G1 and G2, in line with previous studies [13, 14]. In pNENs, a low microvessel density is indicative of a poor prognosis and an elevated risk of metastasis [25]. Well-differentiated pNENs (G1 and G2) with overexpression of the vascular endothelial growth factor receptor and platelet-derived growth factor receptor pathways result in increased neovascular density and local blood supply within the tumour [41], exhibiting hyper-enhancement relative to the pancreatic parenchyma. In contrast, high-grade pNENs are more prone to vascular invasion and microthrombosis than low-grade pNENs, causing local necrosis in the lesion and exhibiting reduced blood supply [42]. Although the V, D are effective indicators for distinguishing between G3 and NEC to G1 and G2 in LASSO regression analysis, the D is excluded in multivariate regression analysis. However, D retains its potential value in the differentiation of pNENs, as mentioned above, NEC can lead to significant tissue fibrosis [36, 37]. While enhancement scans of lesions with abundant interstitial and fibrotic components tend to show delayed enhancement [43]. Therefore, we speculated that D might be informative in distinguishing NEC from G3.

Among other clinical and radiological features, NSE levels, tumour size, and necrosis correlated with the pathological grading of pNENs. G3 and NEC in this study had higher levels of NSE expression, which is similar to a report that NSE is more sensitive for diagnosing poorly differentiated NECs [1]. Furthermore, several studies have discovered that high-grade pNENs have higher NSE expression than do low-grade pNENs [44], and the NSE level correlates with patient prognosis [45]. Our results showed that G3 and NEC had larger tumour diameters than did G1 and G2, which is consistent with the results of a previous study [40]. High-grade pNENs are more prone to necrosis than low-grade pNENs, aligned with previous studies [46]. This is because high-grade pNENs have a higher Ki-67 proliferation index (> 20%) and more rapid cell proliferation. This leads to a dramatic increase in the demand for nutrients and oxygen by tumour cells, and angiogenesis within the tumour is often unable to meet this rapid growth, leading to local ischaemia and hypoxia, which in turn triggers necrosis [47].

According to the “ENETS 2023 guidance paper for nonfunctioning pancreatic neuroendocrine tumours” [6], when NF-pNENs are less than 2 cm with no metastasis or regional lymph node invasion, immediate surgery may be unnecessary, but regular follow-up is required. Our study demonstrated that multifrequency MRE-related parameters (stiffness, fluidity) can non-invasively predict pNENs smaller than 2 cm (N0, M0) as low-grade pNENs, especially G1, which may increase clinicians’ confidence in making the clinical decision to follow up for observation. On the contrary, if the MRE parameters suggest that the lesion may be G3 or NEC, even if the lesion meets the indications for follow-up, surgical resection may need to be carefully considered as an active strategy to improve the long-term outcome. In addition, SSTR expression and Ki-67 > 55% are also very important for the treatment selection and prognosis prediction of pNENs [6, 7]. Therefore, the application of multifrequency MRE in pNENs can be further explored with these biomarkers. It is worth mentioning that the collaboration of MRE with DOTATATE-PET/CT and FDG-PET/CT can further improve the accuracy of differentiating high and low-grade pNENs. In cases where the suspicion is raised of neuroendocrine tumours, despite low uptake of both DOTATATE-PET/CT and FDG-PET/CT scans, if MRE indicates high lesion stiffness, the potential for G3 and NEC should nevertheless be given due consideration, with a view to reducing the incidence of misdiagnosis.

Despite these promising findings, this study had some limitations. First, it was a single-centre study, highlighting the need for including more patients in future multicentre studies and external validation of the results. Second, we did not investigate the potential effects of different MRI field strengths and devices from various manufacturers on the results. Third, the ROIs of the multifrequency MRE didn’t exactly match the pathological assessment. Therefore, in the future, we will try to correlate whole-tumour stiffness and fluidity with whole-tumour pathology by using spatial mapping and volumetric analysis to further explore the role of multifrequency MRE parameters. Finally, this study did not further differentiate between G3 and NEC, and more samples need to be included to clarify the utility of using multifrequency MRE in differentiating between pNENs G3 and NEC.

In conclusion, this study showed that multifrequency MRE parameters improved the predictive efficacy of conventional MRI parameters in predicting the pathological grade of pNENs. The predictive performance of the combined model (V + *c*) was excellent, which means the hypo-enhancement and increased stiffness of the lesion relative to the pancreatic parenchyma in the portal venous phase is highly suggestive of the possibility of G3 and NEC. We suggest that multifrequency MRE can be added to the MRI examination before the treatment of pNENs, only increasing the scanning time of 5–7 min, without increasing the use of contrast media and radiation effects. This may aid in clinical decision-making and prognostic prediction.

## Supplementary information

ELECTRONIC SUPPLEMENTARY MATERIAL

## Data Availability

The original data generated in this study can be requested from the corresponding author on reasonable request. You can contact the corresponding author via email at luoyj26@mail.sysu.edu.cn to discuss the specific conditions for data access and possible measures for privacy protection.

## Abbreviations

A: Relative enhancement degree of lesions during the arterial phase
ADC: Apparent diffusion coefficient
AUC: Area under the curve
CI: Confidence interval
D: Relative enhancement degree of lesions during the delayed phase
MEGs: Motion-encoding gradients
MRE: Magnetic resonance elastography
NEC: Neuroendocrine carcinoma
NET: Neuroendocrine tumours
NSE: Neuron-specific enolase
PDAC: Pancreatic ductal adenocarcinoma
pNENs: Pancreatic neuroendocrine neoplasms
ROI: Region of interest
V: Relative enhancement degree of lesions during the portal venous phase
