# Supplementary material for: Added value of multifrequency magnetic resonance elastography in predicting pathological grading of pancreatic neuroendocrine neoplasms
Source: Insights Imaging. 2025 Jun 12;16:119. doi: 10.1186/s13244-025-02008-3 (PMC12162458; doi:10.1186/s13244-025-02008-3)
Supplement: Supplementary file 1 — ELECTRONIC SUPPLEMENTARY MATERIAL [file 13244_2025_2008_MOESM1_ESM.pdf]

Added Value of Multifrequency Magnetic Resonance Elastography in Predicting Pathological Grading of Pancreatic Neuroendocrine Neoplasms

ELECTRONIC SUPPLEMENTARY MATERIAL

A: Supplementary Text

Material and Methods

Imaging technique

MR sequences and multifrequency MRE

The protocol included axial multiecho Dixon volume-interpolated breath-hold T1-weighted imaging (T1WI), axial and coronal half-Fourier acquisition single-shot turbo spin-echo T2-weighted imaging (T2WI), axial trigger turbo spin-echo fat-saturated (fs) T2WI, fat-suppressed diffusion-weighted imaging with single-shot echo-planar imaging (EPI), dynamic contrast-enhanced time-resolved imaging, and pancreatic multifrequency MRE.

Image evaluation

The evaluation details of these radiological features were as follows: the tumour components included predominantly solid (> 90% solid portion), solid and cystic (10–90% solid portion), and predominantly cystic (< 10% solid portion) [1]. Tumour necrosis was defined as the presence of intratumoural areas exhibiting high signal intensity on T2WI with no contrast enhancement on dynamically enhanced images [2]. The signs of intratumoural haemorrhage observed on MRI were as follows: (1) high intensity on T1WI (acute-subacute phase) [3], (2) fluid-fluid level in the tumour (chronic phase) [4], or (3) very low intensity of haemosiderin on T2WI (chronic phase) [4]. The relative signal intensity of all pancreatic lesions was evaluated and compared with that of the adjacent normal pancreatic parenchyma, and the relative degree of enhancement of lesions during the arterial (A), portal venous (V), and delayed phases (D) (hypo-, iso-, or hyper-enhancement) was determined. The enhancement pattern is mainly divided into the following types: hyper-enhancement in the arterial phase (Type 1) and persistent hypo- or iso-enhancement in all three phases (Types 2 and 3). Arterial phase with hypo- or iso-enhancement included portal hypo-enhancement with gradual delayed hyper-enhancement on delayed phase images (Type 4) and portal hyper-enhancement with hyper- or iso-enhancement on delayed phase images (Type 5) [5].

Clinical and Pathological Characteristics

Histological grading was assessed the histological grading according to the 2017 WHO classification of tumours, with particular emphasis on the mitotic index and Ki-67 proliferation index [6]. In hematoxylin-eosin-stained sections, the mitotic index was evaluated for mitoses in 50 high-power fields (0.2 mm^2 each) in areas of higher density, and was expressed as mitoses per 10 high-power fields (2.0 mm^2). Immunohistochemical staining was performed using streptavidin peroxidase. Ki-67 expression was considered positive when light brown-yellow granules were observed in the cytoplasm of carcinoma cells, with staining intensity exceeding the non-specific background. The Ki-67 proliferation index was calculated by evaluating more than 500 cells in areas of higher nuclear labelling (so-called hotspots). In cases where diagnosis remained uncertain despited combining pathological morphology and the Ki67 proliferation index, additional immunohistochemical markers, including chromogranin A, synaptophysin, somatostatin receptor 2, cytokeratin, TP53, and RB1, were employed to aid in further classification. When the results of mitotic index grading were inconsistent with the Ki-67 grading results, the grading principle was based on the highest grade [6].

Results

ICCs of multifrequency MRE

Moderate to near-perfect agreements were observed for tumour location (κ=1.000, $p < 0.001$), tumour components (κ=0.832, $p < 0.001$), necrosis (κ=0.853, $p < 0.001$), intratumoural haemorrhage (κ=0.786, $p < 0.001$), tumour size (ICC=0.999, 95% CI: 0.999–1.000, $p < 0.001$), and ADC value (ICC=0.925, 95% CI: 0.890–0.949, $p < 0.001$).

Performance of relevant clinical and radiological features and the combined model

We evaluated the predictive performance of eight features selected via the univariate analysis for predicting the pathological grade of pNENs: c (AUC, 0.847; 95% CI: 0.756–0.938; cut-off, 2.57 m/s), φ (AUC, 0.709; 95% CI: 0.562–0.855; cut-off, 1.10 rad), A (AUC, 0.775; 95% CI: 0.663–0.887), V (AUC, 0.806; 95% CI: 0.699–0.912), D (AUC, 0.821; 95% CI: 0.724–0.919), NSE (AUC, 0.592; 95% CI: 0.399–0.785; cut-off, 47.30 ng/ml), tumour size (AUC, 0.692; 95% CI: 0.551–0.834; cut-off, 49.00 mm), and necrosis (AUC, 0.639; 95% CI: 0.522–0.757). The combined model (V+c) (AUC, 0.930; 95% CI: 0.876–0.984) demonstrated the best predictive performance compared with that of c (p = 0.021), φ (p = 0.003), A (p = 0.002), V (p = 0.010), D (p = 0.018), NSE ($p <$ 0.001), tumour size (p = 0.001), and necrosis ($p < 0.001$). (Table S1 and Figure S1).

References:

1. Kim DW, Kim HJ, Kim KW, et al (2014) Neuroendocrine neoplasms of the pancreas at dynamic enhanced CT: comparison between grade 3 neuroendocrine carcinoma and grade 1/2 neuroendocrine tumour. Eur Radiol 25: 1375-1383.
2. Kim H, Kim DH, Song IH, et al (2022) Survival Prediction after Curative Resection of Pancreatic Ductal Adenocarcinoma by Imaging-Based Intratumoral Necrosis. Cancers (Basel) 14.
3. Bonneville F, Cattin F, Marsot-Dupuch K, Dormont D, Bonneville JF, Chiras J (2006) T1 signal hyperintensity in the sellar region: spectrum of findings. Radiographics 26: 93-113.
4. Kurihara N, Takahashi S, Higano S, et al (1998) Hemorrhage in pituitary adenoma: correlation of MR imaging with operative findings. Eur Radiol 8: 971-976.
5. Jeon SK, Lee JM, Joo I, et al (2017) Nonhypervascular Pancreatic Neuroendocrine Tumors: Differential Diagnosis from Pancreatic Ductal Adenocarcinomas at MR Imaging—Retrospective Cross-sectional Study. Radiology 284: 77-87.
6. Lloyd RV, Osamura R, Kloppel G, et al (2017). 4th edition. WHO classification of tumours of endocrine organs, vol. 10. Lyon (France): IARC Press.

B: Supporting Table and Figure

Table S1. Multicollinearity analysis for the eight features

Features	Tolerance	VIF
NSE	0.808	1.238
Tumour size (mm)	0.661	1.512
Necrosis	0.724	1.382
A	0.215	4.645
V	0.125	7.991
D	0.237	4.214
Shear wave speed (m/s)	0.723	1.384
Phase angle (rad)	0.780	1.283

VIF, variance inflation factor; NSE, neuron specific enolase; A, relative enhancement degree of lesions during the arterial phase; V, relative enhancement degree of lesions during the portal venous phase; D, relative enhancement degree of lesions during the delayed phase.

Table S2. Performance of clinical and radiological features and the models for differentiating low-/high-grade pNENs

Variables	Delong's Test	AUC (95% CI)	Ac	Se	Sp	PPV	NPV	Cutoff value
NSE (ng/ml)	**<0.001***	0.592 (0.399–0.785)	0.865	0.450	0.974	0.818	0.871	47.30
Tumour size (mm)	**0.001***	0.692 (0.551–0.834)	0.802	0.450	0.895	0.529	0.861	49.00
Necrosis	**<0.001***	0.639 (0.522–0.757)	0.604	0.700	0.579	0.304	0.88	-
A	**0.002***	0.775 (0.663–0.887)	0.781	0.800	0.776	0.485	0.937	-
V	**0.010***	0.806 (0.699–0.912)	0.812	0.750	0.829	0.536	0.926	-
D	**0.018***	0.821 (0.724–0.919)	0.844	0.650	0.895	0.619	0.907	-
c (m/s))	**0.021***	0.847 (0.756–0.938)	0.812	0.750	0.829	0.536	0.926	2.57
φ (rad)	**0.003***	0.709 (0.562–0.855)	0.688	0.750	0.671	0.375	0.911	1.10
V+c	-	0.930 (0.876–0.984)	0.854	0.950	0.829	0.594	0.984	-

pNENs, pancreatic neuroendocrine neoplasms; AUC, area under the curve; CI, confidence interval; Ac, accuracy; Se, sensitivity; Sp, specificity; PPV, positive predictive value; NPV, negative predictive value; NSE, neuron-specific enolase; A, relative enhancement degree of lesions during the arterial phase; V, relative enhancement degree of lesions during the portal venous phase; D, relative enhancement degree of lesions during the delayed phase; c, shear wave speed (stiffness); φ, phase angle (fluidity).

*Statistically significant ($p < 0.05$).

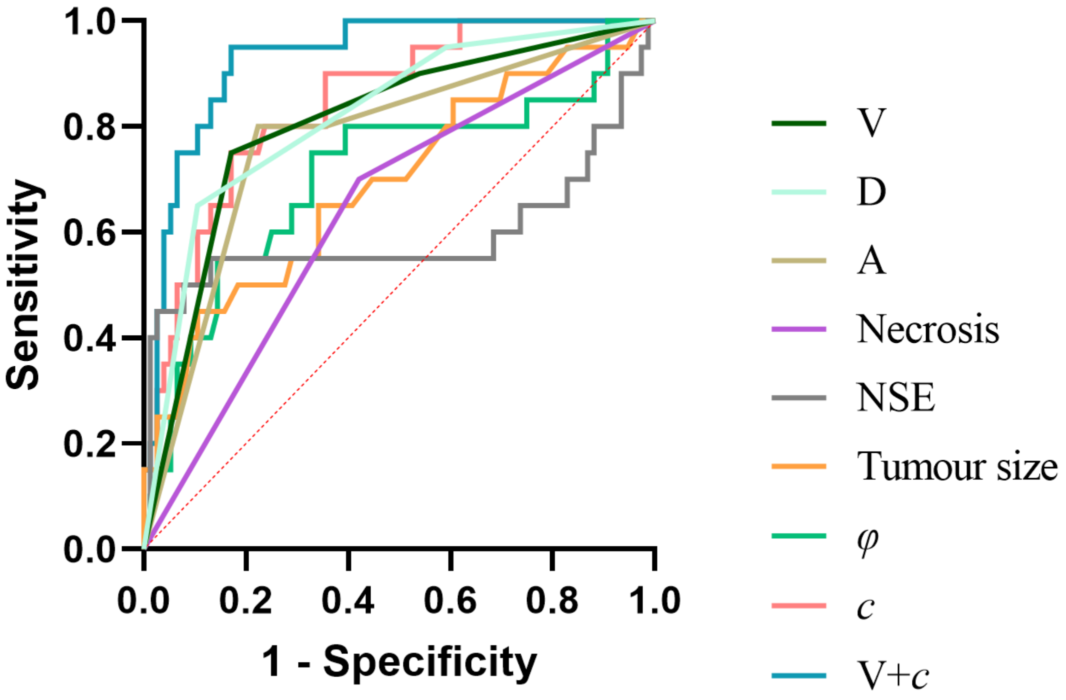

Figure S1. Receiver operating characteristic curves of eight features and model.

The combined model (V+*c*) shows the highest predictive performance (AUC=0.930) compared with that of other clinical (necrosis, NSE) and radiological (V, D, A, tumour size, φ, and *c*) features (All $p < 0.05$).

V, relative enhancement degree of lesions during the portal venous phase; D, relative enhancement degree of lesions during the delayed phase; A, relative enhancement degree of lesions during the arterial phase; NSE, neuron-specific enolase; *c*, shear wave speed (stiffness); φ, phase angle (fluidity).

C: Supporting GIF

The attachments are examples of the actual wave images. The example wave images provided illustrate a single slice from one of the directional measurements, featuring four distinct vibration frequencies (30, 40 50 and 60 Hz). To elaborate, the gif file named "wave_20_1_1" corresponds to the first vibration (X-direction) frequency of 30 Hz in the first direction, captured on the 20th slice of the entire image sequence. Adhering to this consistent naming convention, the gif file named "wave_20_1_2" represents the second frequency of 40 Hz in the first direction (X-direction), also on the 20th slice of the image sequence, and so on. In total, forty layers of images were generated for each lesion, each with X, Y, and Z directions, and each with a waveform at 30, 40, 50, and 60 Hz frequencies, so there were a total of 480 gif files, and we chose the middle 20th layer as an example.
